# Proteomic profiling of neuronal mitochondria reveals modulators of synaptic architecture

**DOI:** 10.1186/s13024-017-0221-9

**Published:** 2017-10-27

**Authors:** Laura C. Graham, Samantha L. Eaton, Paula J. Brunton, Abdelmadjid Atrih, Colin Smith, Douglas J. Lamont, Thomas H. Gillingwater, Giuseppa Pennetta, Paul Skehel, Thomas M. Wishart

**Affiliations:** 1 Division of Neurobiology, The Roslin Institute and Royal (Dick) School of Veterinary Studies, University of Edinburgh, Edinburgh, UK; 2 Euan MacDonald Centre for Motor Neurone Disease Research, University of Edinburgh, Edinburgh, UK; 3 FingerPrints Proteomics Facility, College of Life Sciences, University of Dundee, Dundee, UK; 4 Department of Academic Neuropathology, University of Edinburgh, CCBS, Chancellor’s Building, Little France, Edinburgh, UK; 5 Centre for Integrative Physiology, University of Edinburgh, Hugh Robson Building, Edinburgh, UK

**Keywords:** Synapse, Mitochondria, Proteomics, Neuron, Neurodegeneration

## Abstract

**Background:**

Neurons are highly polarized cells consisting of three distinct functional domains: the cell body (and associated dendrites), the axon and the synapse. Previously, it was believed that the clinical phenotypes of neurodegenerative diseases were caused by the loss of entire neurons, however it has recently become apparent that these neuronal sub-compartments can degenerate independently, with synapses being particularly vulnerable to a broad range of stimuli. Whilst the properties governing the differential degenerative mechanisms remain unknown, mitochondria consistently appear in the literature, suggesting these somewhat promiscuous organelles may play a role in affecting synaptic stability. Synaptic and non-synaptic mitochondrial subpools are known to have different enzymatic properties (first demonstrated by Lai et al., 1977). However, the molecular basis underpinning these alterations, and their effects on morphology, has not been well documented.

**Methods:**

The current study has employed electron microscopy, label-free proteomics and in silico analyses to characterize the morphological and biochemical properties of discrete sub-populations of mitochondria. The physiological relevance of these findings was confirmed in-vivo using a molecular genetic approach at the *Drosophila* neuromuscular junction.

**Results:**

Here, we demonstrate that mitochondria at the synaptic terminal are indeed morphologically different to non-synaptic mitochondria, in both rodents and human patients. Furthermore, generation of proteomic profiles reveals distinct molecular fingerprints – highlighting that the properties of complex I may represent an important specialisation of synaptic mitochondria. Evidence also suggests that at least 30% of the mitochondrial enzymatic activity differences previously reported can be accounted for by protein abundance. Finally, we demonstrate that the molecular differences between discrete mitochondrial sub-populations are capable of selectively influencing synaptic morphology in-vivo. We offer several novel mitochondrial candidates that have the propensity to significantly alter the synaptic architecture in-vivo.

**Conclusions:**

Our study demonstrates discrete proteomic profiles exist dependent upon mitochondrial subcellular localization and selective alteration of intrinsic mitochondrial proteins alters synaptic morphology in-vivo.

**Electronic supplementary material:**

The online version of this article (10.1186/s13024-017-0221-9) contains supplementary material, which is available to authorized users.

## Background

Ageing is a fundamental risk factor for the development of a large range of neurodegenerative diseases, which are characterized by the selective death of neuronal subpopulations [1, 2]. Neurons are highly polarized cells consisting of three distinct functional domains: the cell body, axon and the synapse. Previously, it was believed that the clinical phenotypes of neurodegenerative diseases were caused by the loss of entire neurons [3], however it has recently become apparent that these neuronal sub-compartments can degenerate independently of one another [4, 5], with synapses being particularly vulnerable to a broad range of stimuli. Whilst the properties of the potential differential degenerative mechanisms remain largely unknown, numerous themes have consistently appeared in the literature, suggesting that proteins regulating the ubiquitin-proteasome system [6–8], oxidative stress [9–12] and mitochondria [1, 6, 10, 12–15] may all play a role in regulating the stability of the synaptic compartment.

Synaptic compartments constantly demand ATP to maintain ionic gradients and neurotransmission events [16]. In addition synapses also demonstrate a substantial need for calcium buffering machinery [13]. Accordingly, sub-populations of mitochondria are enriched pre- and post-synaptically [1, 6, 10]. Such synaptic mitochondria are reportedly distinguishable from non-synaptic mitochondria, displaying unique enzymatic [17], calcium buffering [18, 19] and antioxidant properties [10]. However, if and how these distinctive subpopulations of synaptic mitochondria influence the vulnerability of synaptic compartments remains largely unknown.

In an attempt to address this, we have used label-free proteomics to characterise the proteomes of synaptic and non-synaptic mitochondria following established biochemical isolation methods [17]. By utilizing this methodology, we have generated proteomic profiles that reveal consistent molecular fingerprints for synaptic and non-synaptic mitochondria. Quantitative fluorescent western blotting was used to confirm these proteomic and fractionation data in a range of species, including rat and ovine tissues. These results were consistent with the existence of distinct mitochondrial sub-populations containing patterns of relative protein abundances that are conserved between different mammalian species. To determine if the molecular differences between these mitochondrial subpopulations may be capable of influencing the vulnerability of synapses, we manipulated the expression of mitochondrial candidates in vivo to assess synaptic stability in the *Drosophila* neuromuscular system. Our data demonstrate that selective knock-down of intrinsic mitochondrial proteins identified in this manner have the potential to alter synaptic morphology and the area of the presynaptic active zone in vivo. Thus, changes in mitochondrial protein expression may contribute to increased synaptic vulnerability and dysfunctional neurotransmission in early molecular pathological processes during ageing and/or disease.

## Methods

### Ethics

In line with the 3Rs no animals were bred or sacrificed specifically for this project.

### Rats

Nine male wild-type Sprague Dawley rats aged ca. 24 weeks (actual range 168-171 days) were used. Rats were group housed (3-4 per cage) in a SPF facility in open-top cages and maintained on a 12-12 h light-dark cycle (lights on at 07:00 h), under controlled temperature (22 ± 2 °C) and humidity (55 ± 5%) with free access to drinking water and standard 14% protein rodent diet (Harlan Teklad). Rats were killed by conscious decapitation and brains were immediately excised. Brain stem and cerebellum were removed and discarded. Fresh forebrains were weighed and pooled for homogenization before mitochondrial preparations.

### Sheep

Three Scottish blackface female sheep aged 5 years were utilized. Sheep were sacrificed at the Farm Animal Teaching Hospital, Royal (Dick) School of Veterinary Studies, University of Edinburgh by anaesthetisation and exsanguination. Brains were excised, bisected at the sagittal midline and the brain stem and cerebellum were removed and discarded. Tissues were submerged in ice cold high magnesium artificial cerebral spinal fluid (NaCl 125 mM; NaHCO_3_ 26 mM; glucose 25 mM; KCl 2.5 mM; NaH_2_PO_4_(2H_2_O) 1.25 mM; CaCl_2_ 1 mM; MgCl_2_ 4 mM) to maintain brain structural integrity before cortical mitochondrial preparations.

### Mitochondrial preparations

Methodology comprehensively reported in Lai et al. [17] was employed to produce proteomic data comparable to early biochemical reports. All procedures were carried out at 4 °C. Forebrains were homogenized by hand in isolation medium (0.32 M sucrose, 1 mM K-EDTA, 10 mM, Tris HCl) to give a 1:10 homogenate. The homogenate was centrifuged at 1300 g for 3 min and the resulting pellet was manually resuspended in 15 ml isolation medium before recentrifugation as before. Supernatants were pooled and centrifuged at 17,000 g for 10 min to give the crude mitochondrial pellet (CM). The CM pellet was resuspended in 15 ml isolation medium and 5 ml of the suspension was layered into 3 tubes containing 7 ml of 7.5% Ficoll-sucrose medium (7% Ficoll, 0.32 M sucrose, 50 μM K-EDTA, 10 mM Tris HCl) on top of 7 ml of 10% Ficoll-sucrose medium (10% Ficoll, 0.32 M sucrose, 50 μM K- EDTA, 10 mM Tris HCl) and centrifuged at 99,000 g for 30 min on a swing- out Beckman ultracentrifuge. This resulted in a myelin (My) fraction banded at the top, a synaptosomal (Syn) fraction at the second interphase and a ‘free’ (non-synaptic) mitochondrial pellet (M) at the bottom of the tube. The myelin fraction was aspirated and the synaptic fraction collected, without disturbing the pellet. This was then diluted in 3× isolation medium and centrifuged at 18,500 g for 10 min. The non-synaptic mitochondrial pellet was frozen immediately on dry ice before storage at −80 °C. The resulting synaptosomal pellet was lysed and resuspended in 30 ml 6 mM Tris-HCl (pH 8.1) and centrifuged at 11,800 g for 10 min. The supernatant was removed and the pellet was again resuspended in 10 ml of 6 mM Tris-HCl (pH 8.1) and recentrifuged at 8300 g for 10 min. The supernatant was discarded and the pellet resuspended in 10 ml of 3% Ficoll medium (3% Ficoll, 0.12 M mannitol, 30 mM sucrose, 25 μM K-EDTA, 5 mM Tris-HCl).

One third of this suspension was layered into 3 tubes containing 5 ml 4.5% Ficoll medium (4.5% Ficoll, 0.24 M mannitol, 60 mM sucrose, 50 μM K-EDTA, 10 mM Tris-HCl) on top of 10 ml 6% Ficoll (6% Ficoll, 0.24 M mannitol, 60 mM sucrose, 50 μM K-EDTA, 10 mM Tris-HCl) and centrifuged at 11300 g for 30 min in a swing-out Beckman ultracentrifuge. After centrifugation, the top band was removed and the intermediate band decanted and diluted with an equal volume of isolation medium. This lysate was then centrifuged at 17,000 g for 10 min providing synaptosomally derived mitochondria (SM population) and an SM2 fraction (pellet). The SM and SM2 fractions were pooled to give a greater yield for proteomic experiments and validation, frozen on dry ice and stored at −80 °C.

### Protein concentration assay

Samples were homogenized in label-free or RIPA buffer +1% protease cocktail inhibitor (Thermo Scientific). After homogenization, samples were centrifuged at 20,000 g for 20 min at 4 °C. The supernatant containing the solubilized protein was removed and pellets discarded. Protein concentration of samples was determined using a Pierce Micro BCA assay kit according to the manufacturers instructions.

### Label-free proteomics

Synaptic and non-synaptic mitochondrial preparations were extracted in SDT lysis buffer containing 100 mM Tris-HCl (pH 7.6), 4% (*W*/*V*) sodium dodecyl sulfate (VWR) and 0.1 M d/l-dithiothreitol (Sigma). For efficient protein extraction, lysates were freeze–thawed and homogenized in SDT buffer several times. Protein concentration was then determined using BCA assay. Aliquots (2 mg) of each mitochondrial preparation were processed through FASP (filter-aided sample preparation) involving buffer exchange to 8 M urea and alkylation with 50 mM iodoacetamide prior to a double digestion with trypsin (Roche, sequencing grade), initially overnight, then for an additional 5 h at 30 °C. Resulting peptides were desalted then separated using an Ultimate 3000 RSLC (Thermo Scientific) nanoflow LC system. Using an ESI Easy Spray source at 50 °C, technical replicates (3 × 0.75 μg) of each sample were loaded with a constant flow of 5 μL/min onto an Acclaim PepMap100 nanoViper C18 trap column (100 μm inner diameter, 2 cm length; Thermo Scientific). After trap enrichment for 3 min, peptides were eluted onto an Acclaim PepMap RSLC nanoViper, C18 column (75 μm, 50 cm; Thermo Scientific) with a linear gradient of 2–40% solvent B (80% acetonitrile with 0.08% formic acid) over 90 min with a constant flow of 300 nL/min. The HPLC system was coupled to a linear ion trap Orbitrap hybrid mass spectrometer (LTQ-Orbitrap Velos Pro, Thermo Scientific) via a nanoelectrospray ion source (Thermo Scientific). The spray voltage was set to 1.6 kV, and the temperature of the heated capillary was set to 250 °C. Full-scan MS survey spectra (*m*/*z* 335–1800) in profile mode were acquired in the Orbitrap with a resolution of 60,000 after accumulation of 1,000,000 ions. The 15 most intense peptide ions from the preview scan in the Orbitrap were fragmented by collision-induced dissociation (normalized collision energy, 35%; activation *Q*, 0.250; and activation time, 10 ms) in the LTQ after the accumulation of 10,000 ions. Dynamic exclusion parameters were set as follows: repeat count, 1; repeat duration, 30 s; exclusion list size, 500; exclusion duration, 45 s; exclusion mass width, plus/minus 10 ppm (relative to reference mass). Maximal filling times were 1000 ms for the full scans and 150 ms for the MS/MS scans. Precursor ion charge state screening was enabled, and all unassigned charge states as well as singly charged species were rejected. The lock mass (445.120024), option was enabled for survey scans to improve mass accuracy. Data were acquired using the Xcalibur software (for all raw data see PRIDE project accession: PXD005537).

Raw proteomic data were imported into Progenesis for characterization and analysis of relative ion abundance. 2D representations of MS/MS output were created for each sample and these were aligned to determine similar features (average alignment score > 90%). Following alignment, data was filtered by retention time with features detected below 5 min and above 110 min discarded to correct for elution variability and peptides with charge state between 2 and 5 only included in the search. The runs were grouped according to subcellular localization (synaptic and non-synaptic) and Statistical *P* values were automatically generated in Progenesis software through a 1 way ANOVA on the ArcSinh transform of the normalized data.

Peptides were filtered by the following criteria: power < 0.8, fold change >2, *p*<0.05 and the remaining data were exported from Progenesis for identification of individual peptide sequences using the Uniprot Swall subspecies *Rattus norvegicus* via Mascot Search Engine (V2.3.2). Enzyme specificity was set to that of trypsin, allowing for cleavage N-terminal to proline residues. Other parameters used were as follows. (i) Variable modifications: methionine oxidation, methionine dioxidation, protein N-acetylation, gln → pyro-glu. (ii) Fixed modifications: cysteine carbamidomethylation. (iii) MS/MS tolerance: FTMS- 10 ppm, ITMS- 0.6 Da. (iv) Maximum missed cleavages: 2. (vi) False Discovery Rate: 1%. A cutoff score of >29 was used based on Mascot probability threshold of 0.05 that the observed hit is a random event. As an indication of identification certainty, the false discovery rate for peptide matches above identity threshold was set at 1%.

Identified proteins were re-imported into Progenesis for further processing. Proteins were subject to stringent filtering parameters to eliminate those which had <2 unique peptides, <2-fold change between subpopulations and *p*>0.05 to obtain the proteins which demonstrated the largest significant variation in expression between synaptic and non-synaptic mitochondria. See Additional file 1: Table S1 and Additional file 2: Table S2 for raw unfiltered and filtered data respectively.

### Ingenuity pathway analysis

IPA was performed as previously described [20] with the interaction data limited as follows: direct and indirect interactions; experimentally observed data only; 35 molecules per network; 10 networks per dataset. Prediction activation scores (z-score) were calculated in IPA. The z-score is a statistical measure of the match between an expected relationship direction and the observed protein expression. Positive z-scores indicate activation (orange) and negative z-scores indicate inhibition (blue) [21].

### Quantitative fluorescent western blotting

Quantitative fluorescent western blotting was performed as previously described [22]. Samples were diluted to provide desired protein concentration. 10-15 μg protein was loaded per well into Nu-PAGE**®** Novex**®** 4-12% Bis Tris mini-gels (Life Technologies) and transferred to PVDF membranes using an iBlot**®** and Invitrogen gel transfer stacks. Membranes were incubated in primary antibodies at 4 °C and secondary antibodies at room temperature (concentrations according to manufacturers instructions) before imaging on Li-COR Odyssey infrared scanner. Protein expression wasquantified utilising ImageStudio Lite software (Li-COR Biosciences). *Primary antibodies: citrate synthase (OriGene, Cat. TA310356), VDAC1 (Abcam, Cat. ab14734), telomerase (Abcam, Cat. ab32020), HIBCH (Abcam Cat. ab101672), OGDH (ProteinTech Cat: 15,212-1-AP), COXIV (Abcam Cat. ab16056); secondary anitbodies: Goat anti-rabbit IRDye 680 (Odyssey), donkey anti-mouse IRDye 680 (Odyssey).*

### Transmission electron microscopy

Human mixed sex cortical samples (*n* = 4) from patients without neurological phenotypes were prepared for electron microscopy as previously outlined in detail [23]. Briefly, fresh post-mortem samples, stored in 0.1 M PB were trimmed into small cortical blocks and fixed in 4% paraformaldehyde and 2.5% glutaraldehyde in 0.1 M PB for 48 h. Mouse brain samples were prepared as follows: anesthetized WT male C57BL/6 J mice ((*n* = 4) intraperitoneal injection of Ketanest (100 mg/kg) and Rompun (5 mg/kg)) were killed by perfusion fixation with 0.1 M phosphate buffer containing 4% paraformaldehyde and 2.5% glutaraldehyde before removing the brain and immersing it in fixative for a further 12 h. Following fixation, both human and mouse brain samples were washed in 0.1 m phosphate buffer before cutting free floating 70-μm-thick coronal sections on a Vibratome. Sections were postfixed in 1% osmium tetroxide in 0.1 m phosphate buffer for 45 min. Following dehydration through an ascending series of ethanol solutions and propylene oxide, all sections were embedded on glass slides in Durcupan resin. Regions of interest were glued onto a resin block for sectioning. Ultrathin sections (60–70 nm) were cut and collected on Formvar-coated grids (Agar Scientific), stained with uranyl acetate and lead citrate in an LKB “Ultrostainer”, and then quantitatively assessed in a Philips CM12 transmission electron microscope. Negatives taken in the microscope were scanned into an Apple Macintosh G5 computer using an Epson 4870 Photo flat-bed scanner at 600dpi and subsequently processed using Adobe Photoshop. Images from 232 human and 270 murine slices were analysed using the ImageJ measurements function.

### *Drosophila* stocks

Flies were raised on standard cornmeal food at room temperature. Homology of rat gene of interest and *Drosophila* orthologue was determined by input into DIOPT (DRSC Integrative Ortholog Prediction Tool [24]). Off-target effects of candidate lines were assessed using E-RNAi software [25]. Candidate lines exhibited no off-target effects based on genetic mapping data and all displayed 97.21-100% efficiency for targeting the intended gene (Ndufb8 = 97.75%; mitofilin = 97.21%; Vdac1 = 100%; Aldh = 100%). The *elav-Gal4* driver strain (Bloomington *Drosophila* stock center) was used for all experiments. Stocks were obtained from the VDRC (IDs: 21,707, 47,615, 101,336, 30,413) and Bloomington *Drosophila* stock center (Canton-S). Crosses were maintained at 22 °C for 24 h before removal of adults and embryos were incubated in a water bath at 30 °C to increase levels and activity of the Gal4 proteins.

### Immunohistochemistry

Third instar larvae were selected and dissected in PBS (*n* = 8). The dissected larval neuromuscular junctions (NMJs) were fixed in Bouin’s fixative (15:5:1 picric acid, 37% formaldehyde and acetic acid) for 10 min and washed thoroughly in PBT (PBS + 0.1% TritonX-100). Preparations were blocked in PBT + 10% normal goat serum for 2 h then incubated in primary antibody overnight at 4 °C. NMJs were again washed extensively in PBT and incubated in secondary antibody at room temperature for 2 h. Samples were mounted on microscope slides using Vectashield mounting medium (Vector Laboratories) and imaged on a Zeiss confocal microscope. Images were quantified using the Andlauer & Sigrist, 2012 protocol [26] in ImageJ. Briefly, segmented masks of the fluorescent signal were generated for each channel and the dimension and intensity of each particle was quantified. *Primary antibody: Horseradish peroxidase (HRP (1:200)); secondary antibody: anti-HRP goat biotinylated anti-rabbit (1:400). All antibodies from Jackson ImmunoResearch.*

### Statistical analysis

Data were collected in Microsoft Excel and statistical tests were performed in GraphPad Prism 6 software. For all analyses *p*<0.05 was considered statistically significant. Statistical tests used are detailed in the results or figure legends where appropriate.

## Results

### Mitochondrial subcellular localisation dictates organelle morphology in rodents and humans

**Fig. 1:**
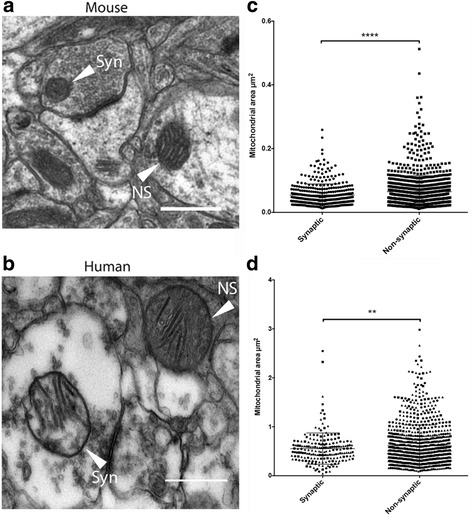
Synaptic and non-synaptic mitochondria are morphologically distinct in both rodent and human brain tissue. **a** and **b**. Example EM images of WT mouse and human cortical tissue displaying synaptic and non-synaptic mitochondria (S = synaptic, NS = non-synaptic). **c** and **d**. Scatterplots representing WT mouse (**c**.) and control human patient (**d**.) cortical synaptic and non-synaptic mitochondrial normalized areas (**c**. synaptic *n* = 391, non-synaptic *n* = 1113 from 4 mice; **d**. synaptic *n* = 177, non-synaptic *n* = 812 from 3 patients). A data point of distinct shape represents each individual animal or patient. Non-synaptic mitochondria appear to be significantly larger than synaptic mitochondria in both mouse and human cortical tissue. Statistical analyses used unpaired two-tailed Student’s t-tests with Welch correction for unequal sample size: ****p<0.0001, **p<0.01). Scale bars = 0.5 μm

Enzymatic activity differences between neuronal mitochondrial sub-populations were first comprehensively described in the 1970s [17]. However, the molecular underpinnings of these biochemical differences and their morphological or physiological consequences were never elucidated. We therefore began by investigating whether mitochondria residing at synaptic terminals were morphologically different from those in non-synaptic compartments. It has previously been suggested that the size of mitochondria may provide indications of biochemical processes as well as neuronal integrity and survival. Numerous studies have begun to describe mitochondrial morphology in the cell body [10, 27–29] but few demonstrate characterisation of those organelles in the axon and synapse – the compartments that show heightened vulnerability to degenerative insult. To establish whether size differences existed between synaptic and non-synaptic mitochondria, we examined transmission electron microscopy images from human and mouse cortical tissue. Mitochondria were classified as ‘synaptic’ if they were <1 μm from the post-synaptic density and vesicles were clearly present within the presynaptic terminal. Mitochondria outside of the pre- and postsynaptic boutons were classed as non-synaptic; anything ambiguous was excluded from the analysis. The results indicate that in both mouse and human cortex, non-synaptic mitochondria appear significantly larger than the organelles present within synaptic terminals ((Fig. 1) mouse: *p*<0.0001; human: *p*=0.0099, respectively). Non-synaptic populations in both species demonstrated elongation versus that of synaptic mitochondria, which displayed a spherical morphology (data not shown). Although these morphological differences between mitochondrial sub-populations may solely reflect adaptations to the particular size and structure of cellular compartments, we hypothesised that biochemical adaptations would also likely exist due to the dynamic interplay between protein expression and organelle configuration (see Picard et al., 2013 for a comprehensive review on mitochondrial morphology and function [30]).

### Label-free proteomics reveals significant mitochondrial heterogeneity

**Fig. 2:**
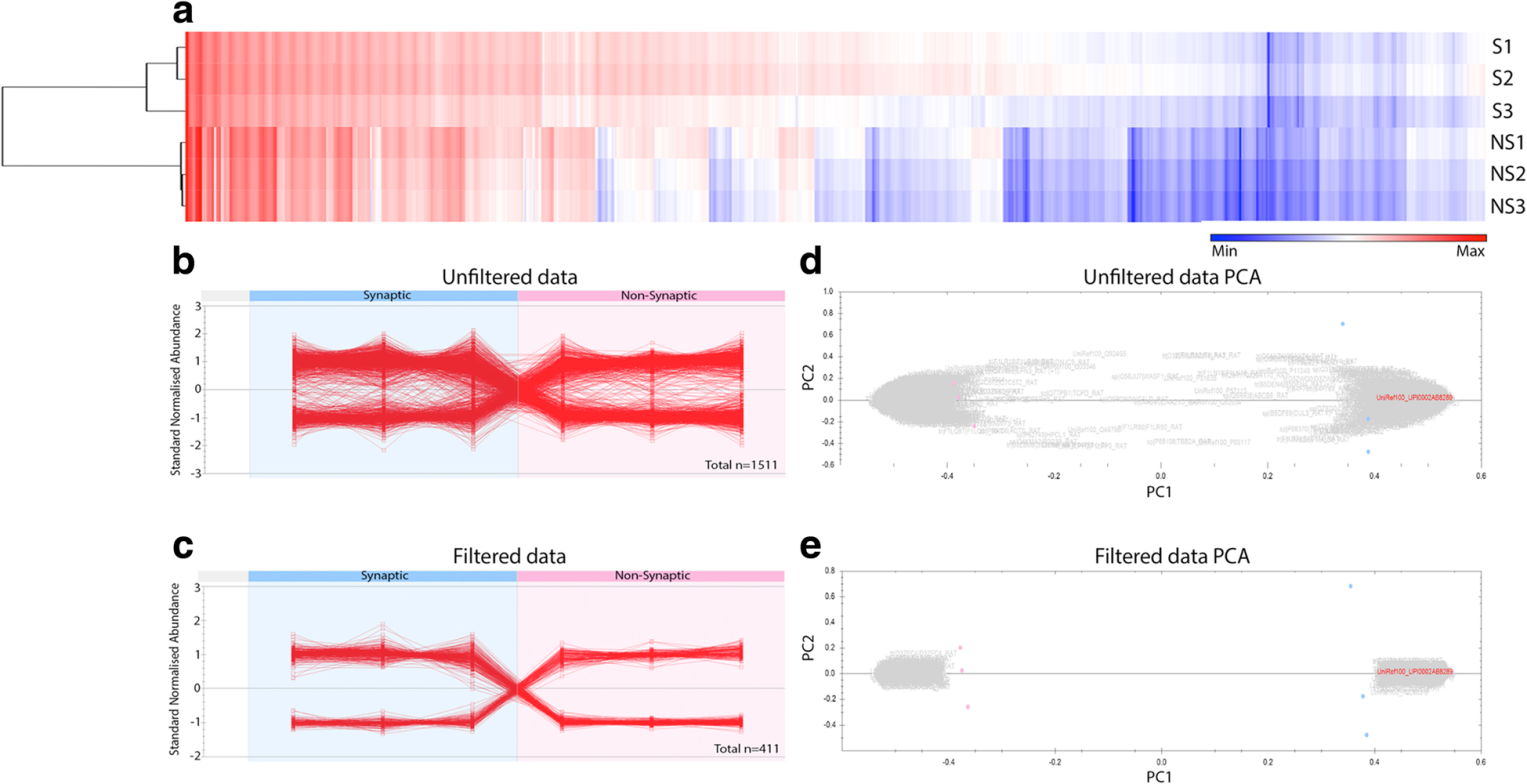
Identification of the mitochondrial proteome. **a**. 2-way clustering of 1511 proteins identified in both synaptic (S) and non-synaptic (NS) mitochondrial samples. Each column represents a single LC-MS/MS run, based on log-transformed data. Proteins (rows) and samples (columns) are clustered by similarities in protein expression intensity, represented by the colour range: dark blue = least abundant, to dark red = most abundant. Dendrogram indicates protein expression differences between synaptic and non-synaptic mitochondrial sub-populations in addition to grouping of individual proteins that demonstrate similar intensity profiles. **b**. Graph represents expression of all 1511 identified proteins with 2 or more unique peptides, independent of magnitude. **c**. Graph displays the 411 proteins which have a > 2 fold change between populations, *p* < 0.05 and were identified with >1 unique peptide. The expression profile demonstrates 2 clear trends of differential protein expression between the sub-populations of mitochondria: proteins that are more abundant in synaptic mitochondria and less abundant in non-synaptic, and vice-versa. **d** and **e**. Principal components analyses of the unfiltered (**d**) and filtered (**e**) proteomic data. Pink data points denote non-synaptic mitochondrial samples and blue data points indicate synaptic mitochondrial samples. Samples are clustered by subcellular localization in both the filtered and unfiltered datasets with little technical variation. Grey displays identified proteins by accession number

To identify molecular differences between mitochondria derived from synaptic and non-synaptic neuronal compartments, we performed label-free proteomics on mitochondrial-enriched fractions isolated from rat forebrain. Quantitative label-free proteomic analyses identified >1500 proteins associated with both synaptic and non-synaptic mitochondria, revealing dynamic variations in protein expression dependent upon subcellular localisation (Fig. 2a and b). Strikingly, over 400 proteins were altered by greater than 2-fold between mitochondrial subpopulations (Fig. 2c), demonstrating significant compartment-dependent biochemical adaptations.

**Fig. 3:**
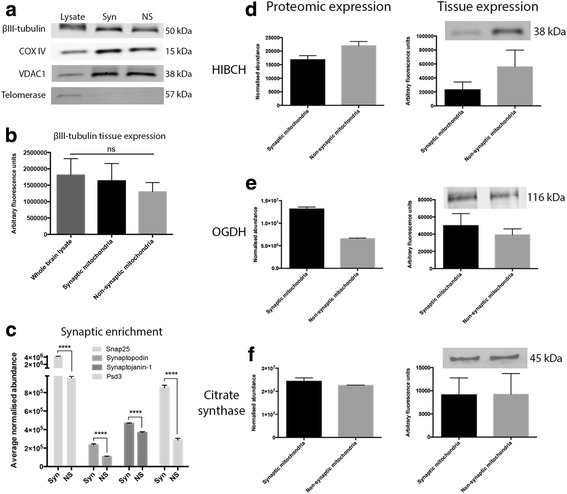
Purification of distinct mitochondrial subpopulations and validation of proteomic data. **a**. Quantitative fluorescent western blots demonstrating relative abundance of the mitochondrial markers COXIV and VDAC1 and the nuclear marker telomerase in whole brain lysate and mitochondrial samples. Mitochondrial samples display little nuclear contamination and enrichment of COXIV and VDAC1. **b**. Quantification of the beta-tubulin loading control signal. **c**. Purity of mitochondrial isolates was verified with quantitative enrichment analyses utilising the raw proteomic data. Comparative expression of the synaptic markers Snap25, Synpo, Synj1 and Psd3 indicate significant enrichment (****p<0.0001) of the proteins in synaptic mitochondrial preparations. **d**-**f**. Left bar chart displays the proteomic average normalised expression values of proteins in synaptic and non-synaptic mitochondria. Right bar chart demonstrates sample protein expression quantified by fluorescent western blots. Proteomic and sample expression of all proteins (HIBCH, OGDH and citrate synthase) follow the same trend thereby providing validation of the proteomic data. Statistical analyses utilized unpaired two-tailed Student’s t-test, n = 3 (** p=<0.01; *** p=<0.001; **** p=<0.0001)

Relative purity of mitochondrial isolates was verified using bioinformatics (Table 1), quantitative enrichment analyses utilizing the raw proteomic data and quantitative fluorescent western blotting (QFWB) on ovine forebrain. The mitochondrial yield obtained from rat forebrain was not sufficient for validation purposes (see methods). The mitochondrial markers VDAC1 and COXIV indicated significant enrichment in synaptic and non-synaptic mitochondrial fractions versus whole brain lysate suggesting purification of the mitochondria from their respective subcellular compartments (Fig. 3a). To demonstrate the purification of mitochondria from discrete neuronal compartments, we calculated the normalised average abundance of the well-established synaptic markers Snap25, Synaptopodin (Synpo), Synaptojanin-1 (Synj1) and Psd3 for both the synaptic and non-synaptic mitochondrial preparations. Indeed, expression levels of Snap25, Synpo, Synj1 and Psd3 indicated significant enrichment in synaptic mitochondrial preparations versus non-synaptic suggesting purification of synaptically derived mitochondria (Fig. 3c). In addition to demonstrating purification of mitochondrial subpopulations, we again employed QFWB to determine the veracity of the proteomic data. We observed corresponding protein expression trends in each tissue preparation, as indicated by the proteomics, for multiple proteins (Fig. 3d-f). Taken together, these results indicate the relative purity of the mitochondrial isolates and suggest the proteomic data is representative of the molecular alterations occurring in the tissue samples. Furthermore, the data indicate that the observed heterogeneity in mitochondrial protein expression may be conserved between mammalian species.

**Table 1:** DAVID 6.7 (NIAID/NIH) enrichment analysis of synaptic and non-synaptic mitochondrial samples. Table comprises the top gene ontology (GO) terms associated with 1511 proteins identified in synaptic and non-synaptic mitochondrial samples. The analysis displays predominantly mitochondrial components, suggesting the samples are enriched for mitochondria

Annotation	Enrichment Score 36.31	Count	*P*_Value	Fold Change	Benjamini
GOTERM_CC_FAT	Mitochondrion	231	2.50E-61	3.00E + 00	1.20E-58
GOTERM_CC_FAT	Mitochondrial part	138	4.50E-54	4.40E + 00	1.10E-51
GOTERM_CC_FAT	Mitochondrial membrane	109	1.70E-47	5.00E + 00	2.60E-45
GOTERM_CC_FAT	Mitochondrial envelope	111	4.20E-46	4.70E + 00	5.00E-44
GOTERM_CC_FAT	Mitochondrial inner membrane	93	7.40E-44	5.40E + 00	7.10E-42
GOTERM_CC_FAT	Organelle inner membrane	94	3.20E-42	5.20E + 00	2.50E-40
GOTERM_CC_FAT	Organelle membrane	164	4.80E-38	2.90E + 00	3.30E-36
GOTERM_CC_FAT	Organelle envelope	118	1.10E-35	3.60E + 00	6.50E.34
GOTERM_CC_FAT	Envelope	118	2.30E-35	3.50E + 00	1.20E-33
KEGG_PATHWAY	Oxidative phosphorylation	57	2.10E-31	6.20E + 00	3.00E-29
SP_PIR_KEYWORDS	Mitochondrion	104	6.80E-30	3.40E + 00	9.70E-28
KEGG_PATHWAY	Parkinson’s disease	54	2.10E-27	5.70E + 00	1.50E-25
KEGG_PATHWAY	Huntington’s disease	59	1.00E-24	4.70E + 00	4.90E-23
KEGG_PATHWAY	Alzheimer’s disease	58	5.40E-23	4.40E + 00	1.90E-21
SP_PIR_KEYWORDS	Mitochondrial inner membrane	35	6.40E-15	4.80E + 00	2.70E-13

### Expression profiling highlights bioenergetic alterations between synaptic and non-synaptic mitochondrial proteomes

**Fig. 4:**
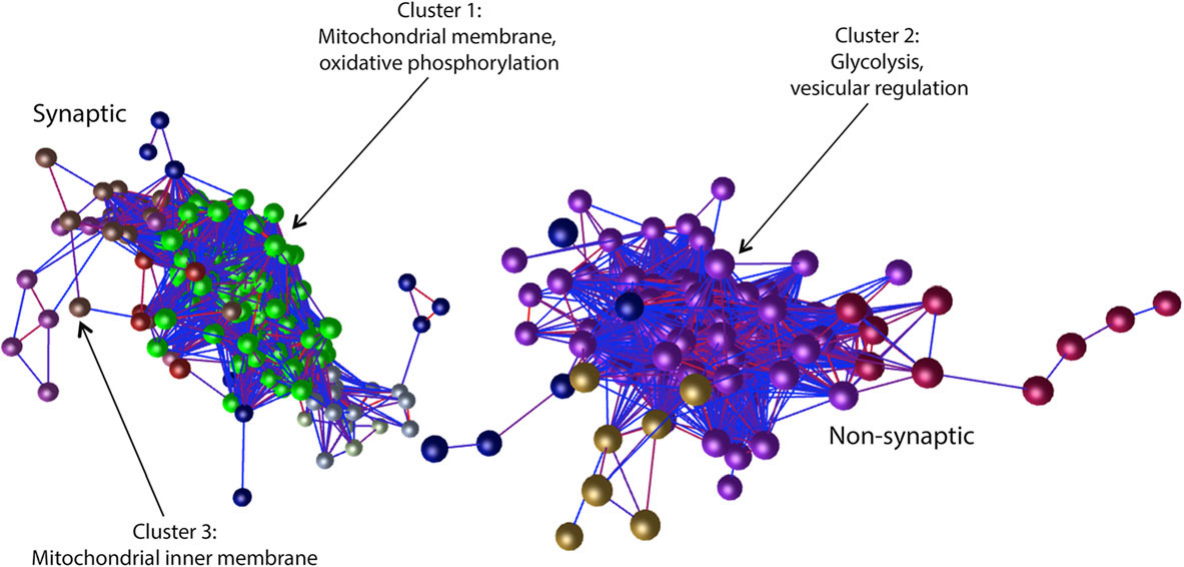
Expression profile clustering identifies alterations associated with bioenergetic control. Nodes (spheres) represent individual proteins and edges (lines) reflect the strength of correlation of expression between proteins. The schematic displays 2 distinct protein networks – proteins displaying increased abundance in synaptic mitochondria on the left and those exhibiting enhanced expression in non-synaptic mitochondria on the right. Coloured nodes represent protein clusters based on expression profile and each cluster displays their representative functions determined by DAVID. *Graph clustered by Pearson r = 0.98*

Having confirmed the veracity of the proteomic data, we next sought to further dissect the proteomic data using Biolayout Express^3D^ [31]. The software applies unbiased Markov clustering algorithms to the input data and groups proteins displaying similar expression trends. This allows visualisation of spatial profiles promoting the identification of physiological cascades altered within the dataset. The nodes, shown as spheres with identical dimensions, signify individual proteins and the edges, represented by connecting lines, are indicative of the correlation of protein expression within the data. Graphs were constructed in 3D space utilizing the 1511 identified proteins from the mitochondrial proteomic analysis (see Fig. 2b and c), generating 10 protein clusters. In agreement with the principal component analysis correlation graphs (Fig. 2d and e), network clustering of the data displayed similar trends with regards to fragmentation of the graph into two localisation-dependent networks (Fig. 4). The fragmentation of the dataset into 2 distinct groups suggests that significant heterogeneity in protein expression exists between the mitochondrial populations derived from discrete neuronal sub-compartments. Indeed, with further examination we exhibited that proteins with increased abundance in synaptic mitochondria cluster into the distinct network on the left of the graph whereas those with increased abundance in non-synaptic mitochondria are observed in the right network (Fig. 4). To identify the functional cascades associated that appear to demonstrate differential expression between the two mitochondrial subpopulations, the top 3 protein clusters were entered into Database for Annotation, Visualization and Integrated Discovery (DAVID) v6.7 to determine the basic functions of the clustered proteins (Fig. 4). Interestingly, the proteins that exhibit upregulation in synaptic mitochondria (clusters 1 and 2) are associated with the inner membrane bioenergetic complexes, which suggests that distinct synaptic and non-synaptic mitochondrial protein expression may reflect altered local requirements for energetic expenditure. Interestingly, these data correlate with the original biochemical study by Lai et al. [17], which reported variations in mitochondrial enzymatic activities dependent upon the subcellular localisation of the organelles. Here, we can account for approximately 35% of those reported enzymatic activity alterations, which are likely due to the differing abundance of particular enzymes in the discrete sub-pools of mitochondria.

### Pathway analysis highlights mitochondrial complex I expression differences as potentially conserved regulators in a range of neurodegenerative conditions

**Fig. 5:**
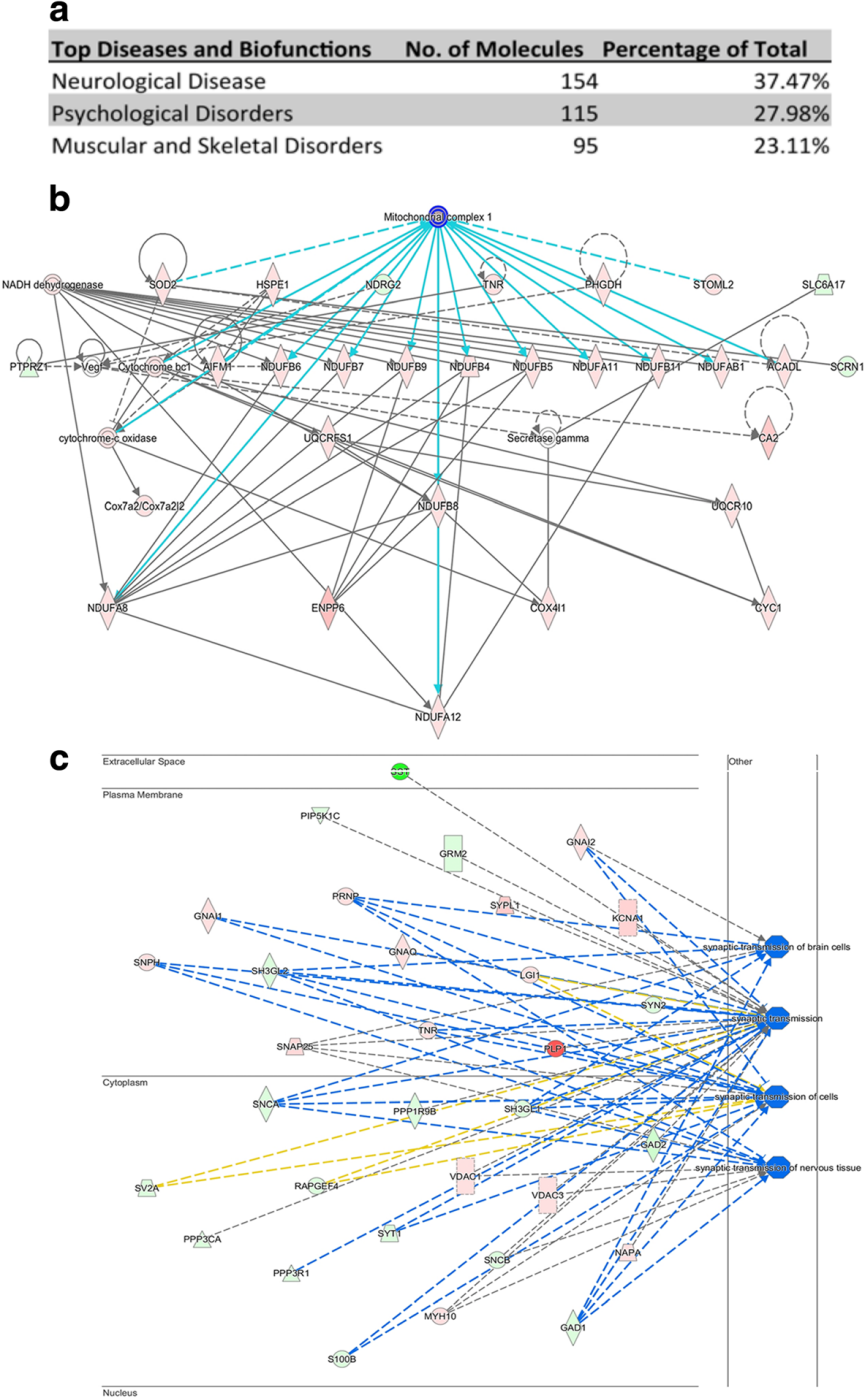
Pathway analysis highlights mitochondrial alterations associated with altered synaptic function and a broad range of neurodegenerative diseases, mediated by complex 1. **a**. 411 differentially expressed mitochondrial proteins are implicated in the literature as contributors to neurological disease, psychological disorders and skeletal and muscular disorders. **b**. Mitochondrial complex 1 is central to the network of interactions (highlighted in light blue) and features a number of intrinsic mitochondrial proteins and 2 protein families. Expression of the synaptic mitochondrial proteome is associated with perturbed synaptic structure and function. **c**. The significant differential expression of proteins in synaptic and non-synaptic compartments appears to inhibit downstream pathways associated with synaptic transmission. *Solid lines indicate direct interactions; dashed lines represent indirect interactions; proteins highlighted in pink are those which are more abundant in synaptic mitochondria; proteins in green are those which are more abundant in non-synaptic mitochondria; dark blue represents predicted pathway inhibition. Shapes are indicative of protein function: diamond = enzyme; rectangle = GPCR; square = cytokine; trapezoid = transporter; circle = other*

In order to determine how differential protein expression in mitochondrial subpopulations may synergistically regulate downstream molecular pathways and cellular processes, we performed an in silico analysis using Ingenuity Pathway Analysis (IPA) software. The software promotes the identification of statistically significant functional groups of proteins, based on known protein interactions and biological functions reported in the published literature [20]. Functional networks generated by IPA software are statistically ranked according to a score calculated via a right-tailed Fischer’s exact test, taking into account the number of original input proteins and the size of the resulting network. With input of the 411 differentially expressed proteins from the mitochondrial proteomic data (>2-fold change), the analysis revealed that 154 (37%) candidates have previously been associated with neurological diseases in the published literature (Fig. 5a), suggesting that a multitude of these proteins may play a role in regulating synaptic stability.

Further examination into the molecular cascades that may be associated the pathogenesis of neurodegenerative diseases highlights that mitochondrial complex I is an upstream regulator of degenerative processes (Fig. 5b). The particular expression of proteins downstream of complex I in synaptic, but not non-synaptic, mitochondria have previously been implicated in numerous neurodegenerative diseases including Huntington’s disease, Parkinson’s disease and Lewy body dementia - all of which demonstrate synaptic perturbations early during pathogenesis (*for a comprehensive review see* [1]). These results suggest that modulation of the synaptic mitochondrial proteome may synergistically drive the demise of the synapse in age-related neuropathologies. In conjunction, assessment of the downstream physiological cascades modulated by synaptic mitochondrial protein expression identified significant inhibition of synaptic transmission cascades (Fig 5c). The increased expression of alpha-synuclein, GAD1, SH3GL1, SH3GL2, S100B PPP3R1 and SYT1 in the non-synaptic mitochondrial proteome appears to indirectly inhibit synaptic transmission. Conversely, the enhanced expression of SNPH, PRNP, GNAI1 and GNAI2 in synaptic mitochondria inhibits neurotransmission cascades in the synaptic compartment. This suggests that heterogeneity in discrete mitochondrial proteomes and their associated downstream pathways has the propensity to promote functional alterations at the synaptic terminal.

### Mitochondrial proteins regulate synaptic morphology in vivo

Although we had generated a compendium of the molecular differences present between synaptic and non-synaptic mitochondrial populations, it remained unclear if any of the identified proteins were capable of actively modulating synaptic stability in vivo. To assess this, we employed a molecular genetic approach using the *Drosophila* larval neuromuscular junction (NMJ) to screen individual proteins to examine their potential role in regulating synaptic morphology. The larval NMJ is an excellent model system to unravel the molecular mechanisms underpinning synaptic structure, function and plasticity [32]. Fundamental mammalian biological and neurological pathways demonstrate conservation in *Drosophila* and recent evidence has indicated that up to 75% of human disease related genes exhibit a functional orthologue in the fruit fly [33]. Larval NMJs harbour glutamatergic synaptic boutons with contiguous invaginated post-synaptic membranes [34, 35], which display significant homology to those found in the mammalian central nervous system. Neurotransmission between these connections has the propensity to invoke plastic modifications [32, 34, 36], providing physiologically relevant mechanistic insights into proteins regulating the structure and function of the synapse in mammalian cognition [36].

**Fig. 6:**
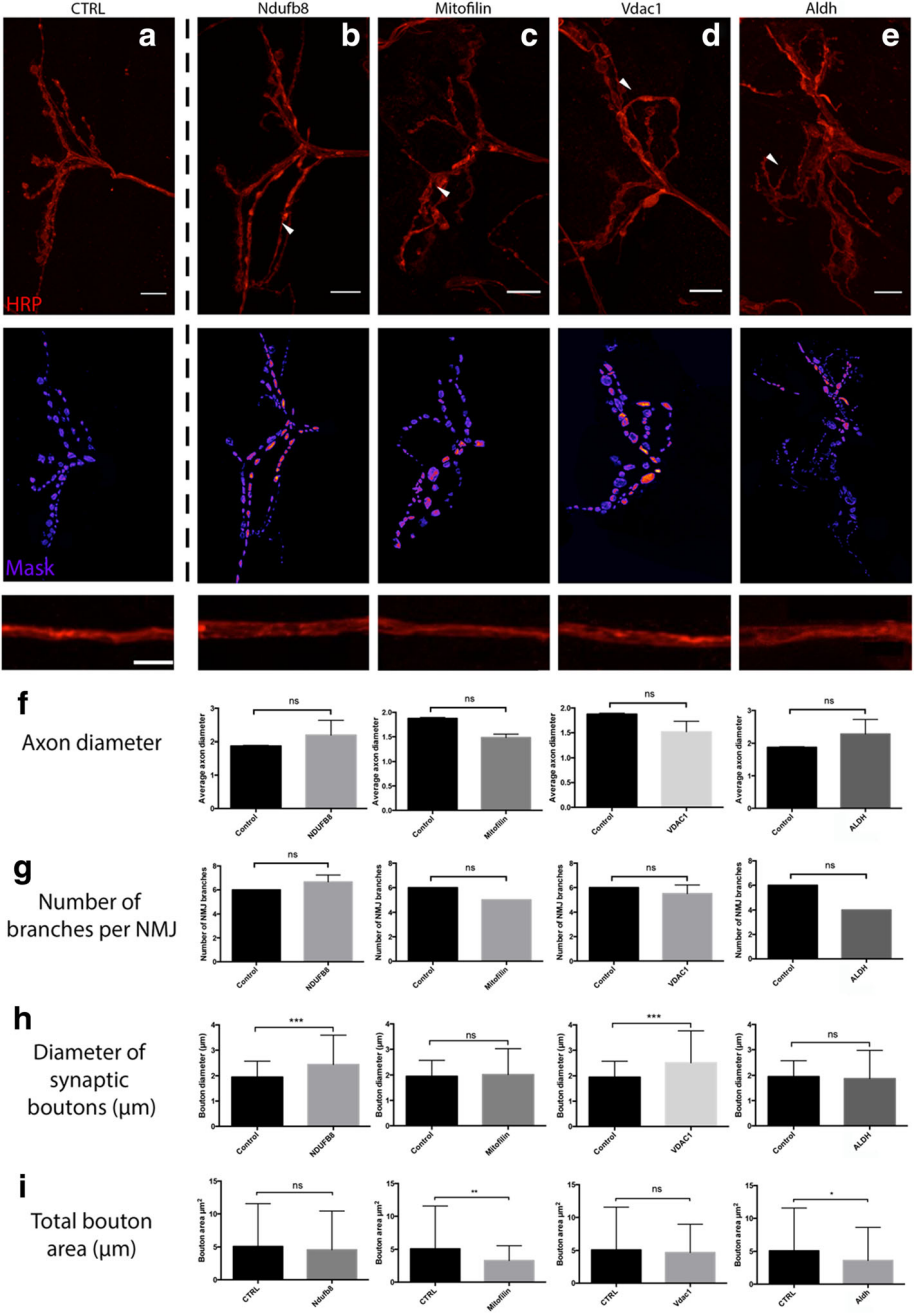
Mitochondrial candidates modulate synaptic morphology in vivo. **a**. Representative image of WT muscle 12 NMJ in abdominal segment A3 (*n* = 8) immunostained with anti-HRP. **b-e**. Top panel displays representative images of muscle 12 NMJ, hemisegment A3 bouton morphology with candidate knock-down. Middle panel demonstrates masks utilized for quantification in ImageJ. Lower panel shows a section of axon. *Scale bar = 10um* for synaptic HRP and mask layers, *5um* for axons. **b**. elav-GAL4/Ndufb8RNAi; **c**. elav-GAL4/MitofilinRNAi; **d**. elav-GAL4/Vdac1RNAi; **e**. elav-GAL4/AldhRNAi. Arrows represent loss or alteration of boutons and/or fragmentation of dendritic branches. A variety of phenotypes exist. *All NMJs immunostained with anti-HRP and imaged at 63× (n = 8).* **f**. Quantification of axon diameter in candidate lines versus control (Control mean ± SEM = 1.88 ± 0.02; Ndufb8 *p*=0.4095, mean ± SEM = 2.19 ± 0.26; Mitofilin *p*=0.169, mean ± SEM = 1.52 ± 0.05; Vdac1 *p*=0.1427, mean ± SEM = 1.52 ± 0.15; Aldh *p*=0.3138, mean ± SEM = 2.28 ± 0.26). **g**. Quantification of number of NMJ branches in candidate lines versus control (Control mean ± SEM = 6.0 ± 0.0; Ndufb8 *p*=0.1161, mean ± SEM = 6.66 ± 0.33; Mitofilin *p*=0.117, mean ± SEM = 5.33 ± 0.33; Vdac1 *p*=0.2722, mean ± SEM = 5.5 ± 0.5; Aldh *p*=0.0522, mean ± SEM = 5.0 ± 0.0). **h**. Quantification of the synaptic bouton diameter in candidate lines versus control (Control mean ± SEM = 1.95 ± 0.06; Ndufb8 *p*=0.0003, mean ± SEM = 2.43 ± 0.1; Mitofilin *p*=0.5759, mean ± SEM = 2.02 ± 0.09; Vdac1 *p*=0.0003, mean ± SEM = 2.51 ± 0.15; Aldh *p*=0.5291, mean ± SEM = 1.87 ± 0.1). **i**. Quantification of the total bouton area in candidate versus control lines (Control mean ± SEM = 5.08 ± 0.47; Ndufb8 *p*=0.4246, mean ± SEM = 4.57 ± 0.44; Mitofilin *p*=0.0078, mean ± SEM = 3.3 ± 0.23; Vdac1 *p*=0.6083, mean ± SEM = 4.68 ± 0.47; Aldh *p*=0.0337, mean ± SEM = 3.6 ± 0.46). All quantification used students t-test; * = p<0.05, ** = p<0.01, *** = p<0.001, **** = p<0.0001

**Fig. 7:**
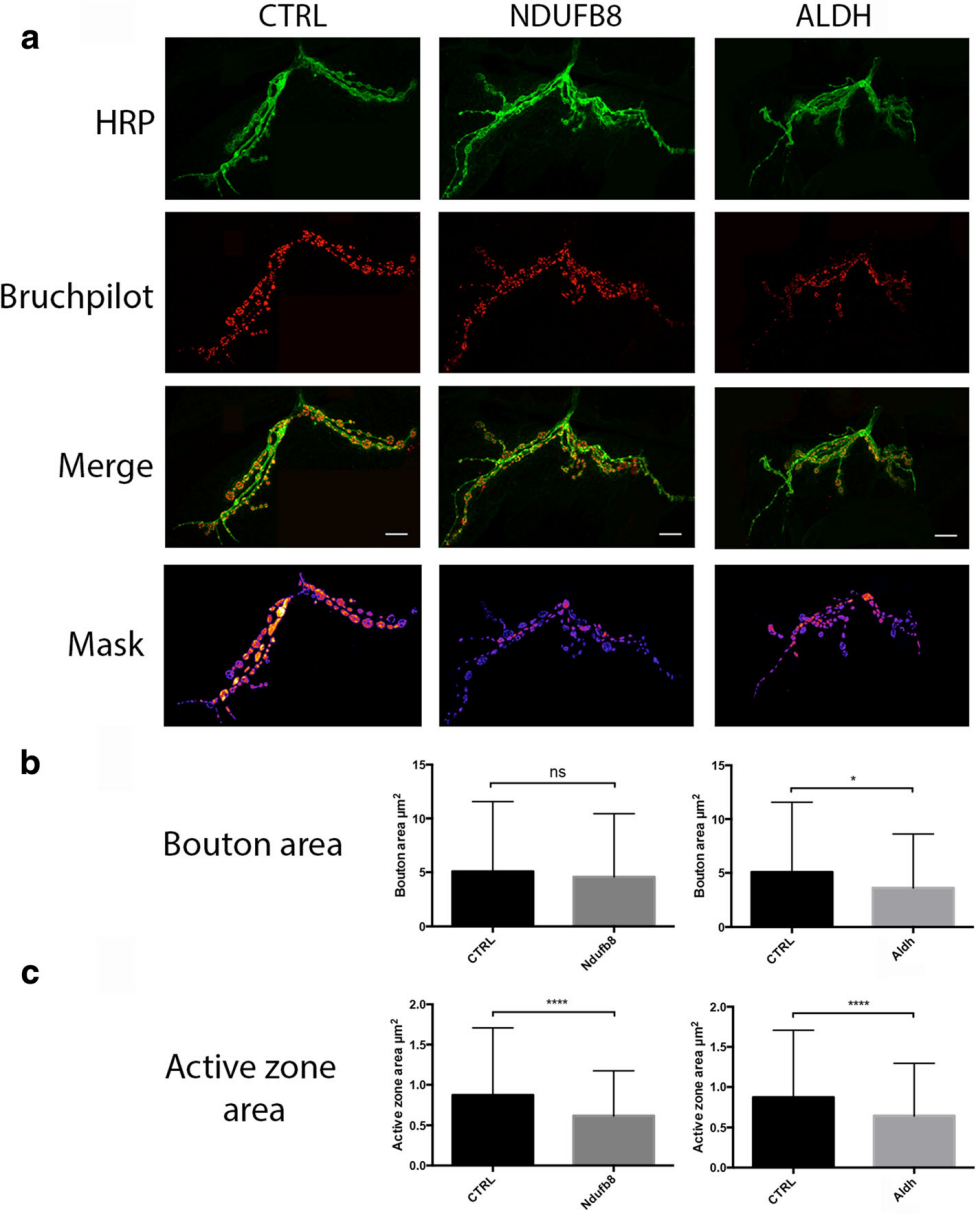
Manipulation of the mitochondrial proteome modulates active zone expression. **a**. Representative images of WT, NDUFB8 and ALDH muscle 12 NMJs from abdominal segment A3 (*n* = 8) immunostained with anti-HRP and anti-bruchpilot. Bottom panel displays ImageJ quantification mask utilized for HRP staining. **b**. Quantification of total bouton area in NDUFB8 and ALDH RNAi lines (as displayed in Fig. 6i). **c**. Quantification of total NMJ active zone area in NDUFB8 and ALDH RNAi lines (Control mean ± SEM = 0.87 ± 0.04; Ndufb8 *p*<0.0001, mean ± SEM = 0.62 ± 0.03; Aldh *p*<0.0001, mean ± SEM = 0.64 ± 0.04. All quantification used students t-test (n = 8); * = p<0.05, ** = p<0.01, *** = p<0.001, **** = p<0.0001. Scale bar = 10um

In order to assess whether the expression of particular proteins in synaptic mitochondria may be regulating the morphology of the synapse, we utilised the *Drosophila UAS*/Gal4 system, which allows tissue specific expression of a particular transgene with use of selected drivers [37]. We selected candidates based on magnitude of change, mitochondrial localisation and availability of *Drosophila* orthologues (see Table 2). Interestingly, the short-listed candidates displayed increased protein expression in synaptic versus non-synaptic mitochondria. Thus, we aimed to selectively knock-down the expression of these mitochondrial proteins in third instar larva neurons for assessment of associated phenotypes. Pan-neural expression of the RNAi constructs (v30413; v47616; v10136; v21707) under control of the *elav-Gal4* driver resulted in viable larva from all crosses. To assess synaptic morphology and potential mitochondrial-dependent alterations in synaptic transmission, we examined the larval muscle 12/13 NMJ by immunohistochemistry with antibodies against the presynaptic marker horseradish peroxidase (HRP) and active zone marker bruchpilot (BRP). Here, we found that the selective RNAi-mediated knock-down of single candidates produced striking synaptic phenotypes at the NMJ (Fig. 6). NDUFB8, mitofilin, VDAC1 and ALDH demonstrated varied phenotypes at the NMJ with alterations in distinct synaptic parameters including bouton diameter and total bouton area (Fig. 6). The most severe phenotype was associated with selective knock-down of aldehyde dehydrogenase (ALDH), which promoted significant reductions (*p*<0.05) in synaptic bouton area with concomitant loss of distinguishable Ib and Is boutons (Fig. 6a). In conjunction, there was a significant decrease (*p*<0.0001) in synaptic bouton active zone staining (Fig. 7a) relative to control NMJs. Similarly, knock-down of neuronal NDUFB8 expression promoted aberrant NMJ morphology (Fig. 6a and b) with significant increases in bouton diameter. Despite this, the NMJs demonstrated a corresponding reduction in active zone area (*p*<0.0001) versus controls (Fig. 7a and c). Interestingly, we did not observe any obvious perturbations in axonal branching or morphology in our *elav-Gal4*/RNAi lines (Fig. 6f and g), suggesting that selective knock-down of these candidates promotes selective alterations in synaptic morphology, mediated by mitochondrial protein expression. Significant reductions in the area of active zone staining in both the ALDH and NDUFB8 RNAi lines suggests that mitochondria may have a direct functional impact on vesicular release and bouton firing properties. Although the mechanistic pathways modulating these alterations remain elusive, regulation of the mitochondrial proteome appears to be significant mediator of synaptic-specific structural and biochemical properties.

**Table 2:** Diopt homology score for Drosophila lines employed in all experiments. Scores demonstrate the degree of homology between the rat gene of interest and equivalent Drosophila orthologue. A score of >2 is considered sufficiently homologous. *Scores range from 1 to 11*

Gene	Fly Gene	Stock	Diopt Score	Magnitude of Change
Ndufb8	CG3192	v30413	8	2.04
Mitofilin	CG6455	v47615	6	1.9
Vdac1	CG6647	v101336	7	2.23
Aldh	CG3752	v21707	5	3.29

## Discussion

The current study has demonstrated novel insights into how a ‘top-down’ approach may be utilised for identifying individual proteins that may modulate the stability of synaptic compartments in vivo. By using a range of techniques we have shown that neuronal mitochondria derived from synaptic and non-synaptic cellular compartments display significant morphological and biochemical differences. Electron microscopy analyses from mouse and human cortical tissue suggest that non-synaptic mitochondria are significantly larger than those found in the synaptic terminal. Alongside these morphological alterations, we have also demonstrated that upwards of 400 mitochondrial associated proteins identified from our label-free proteomic experiments display a ≥ 2-fold expression difference between sub-populations. Using molecular genetic tools at the *Drosophila* neuromuscular junction, we were able to manipulate the expression of 4 of the identified candidates that displayed increased abundance in synaptic mitochondria, resulting in selective alterations in synaptic, but not obvious axonal morphology in vivo. Our data promote further understanding of the basic biology of the mitochondria and how these organelles may regulate the vulnerability of the synapse in a physiologically relevant model. In addition, the results presented may provide increased insight into how mitochondrial perturbations influence the synaptic alterations commonly occurring during ageing and disease.

Our initial proteomic screen revealed a remarkably large and heterogeneous list of candidates that significantly differed in expression dependent upon mitochondrial subcellular localisation. Despite this heterogeneity, upon further analysis of the data utilising in silico tools, it became apparent that many of these proteins have previously been associated with perturbations in synaptic transmission. Experimental manipulation of 4 of these candidates produced varied synaptic phenotypes. The differing morphological phenotypes likely reflect the functional specificities, biological roles and downstream cascades associated with the proteins. NDUFB8, a mitochondrial complex I subunit, produced a relatively mild phenotype at the NMJ with morphological overgrowth but retention of branching patterns seen in controls. The preservation of numerous morphological characteristics despite NDUFB8 knock-down may reflect a compensatory mechanism occurring within the elaborate multi-subunit complex I. Conversely, reductions in ALDH expression resulted in the most severe phenotype, with significant alterations in synaptic bouton morphology and striking alterations within the NMJ architecture. This is likely due to its primary role in protecting mitochondria from endogenous aldehydes generated by lipid peroxidation [38]. With reductions in ALDH, it is probable that an accumulation of noxious metabolites occurs, resulting in cytotoxicity. In support of our observations, a previous study from our laboratory [39] reported that *Drosophila* harbouring mutant ALDH displayed spontaneous degeneration of axons in the olfactory receptor neuron, however the presence of a synaptic phenotype was not morphologically assessed in that system. Furthermore, there have been several recent reports documenting a potential detoxification role for ALDH specifically in populations of dopaminergic neurons [40]. Notably, sub-populations of dopaminergic cells from the brains of Parkinson’s disease patients demonstrated a significant reduction in ALDH [41], which may contribute to alterations in synaptic stability resulting in the activation of degenerative cascades.

Despite NDUFB8 and ALDH demonstrating varied synaptic bouton phenotypes, both candidates promoted a significant reduction in the active zone area. Mitochondrial morphology and function has been associated with alterations in the efficacy of the presynaptic active zone in numerous investigations, however the molecular mediators regulating such events remain elusive. Synaptic transmission is a bioenergetically demanding process requiring the persistent aerobic production of ATP and buffering of intracellular Ca^2+^ concentrations. Evidence indicates that mitochondria redistribute and bind to the active zone in response to synaptic transmission for homeostatic regulation at the synapse [42, 43], promoting the controlled exocytosis underpinning neurotransmission. Disruptions in the mitochondrial proteome may have the propensity to alter the functional organisation and stability of the active zone by reductions in calcium buffering, ATP availability and/or vesicular endocytosis. Although we directly manipulated the expression of NDUFB8 and ALDH, it is likely that the NMJ mitochondria harboured further disruptions to the proteome that were hierarchically driven by alterations in the candidates, which may have synergistically promoted aberrations at the active zone.

Although a conventional RNAi screen would require multiple RNAi and point mutations to be certain of the importance of specific candidates, we have presented several novel synaptic specific phenotypes with selective knock-down of single mitochondrial candidates. However, it is unlikely that the differential expression of all of these proteins can solely regulate synaptic morphology and stability. Instead, it is probable that multiple cellular and molecular pathways, up-and downstream of the mitochondria, converge to modulate compartmental stability in mammalian systems. Thus, differentially expressed mitochondrial candidates and/or their downstream cascades warrant further in vivo investigation to examine the nature and extent of their role in the modulation of synaptic architecture. Despite this, protein expression within the mitochondria, as well as the subcellular localisation of the organelle, are clearly important variables that must be considered when characterising cascades negatively affecting synaptic morphology, transmission and physiology.

A next logical step in determining how the mitochondrial proteome may be contributing to synaptic vulnerability in vivo, is to perform an ageing study using the same tools and techniques presented in the current investigation. It has been well documented that mitochondria dynamically alter dependent on their environment [10, 17–19], and we have provided further evidence for this hypothesis here. However, few studies have attempted to investigate how the synaptic mitochondrial proteome alters during ageing and how variations in protein expression may impact on the function and architecture of the synapse in a physiologically relevant model. An examination of such processes provides scope to potentially identify mitochondrial proteins that may be contributing to synaptic demise during normal healthy and pathological ageing. Furthermore, these data may indicate why such large disparities appear to exist with regards to the vulnerability of synaptic and neuronal populations with different pathogenic insults.

Although the use of label-free proteomics has provided a fairly comprehensive coverage of the mitochondrial proteome (this study yields the highest identification of mitochondrial proteins in a single in vivo analysis to date (1511)), for improved detection of low molecular weight proteins other techniques such as tandem mass tagging with high fractionation may be employed for future experiments. It is highly likely that numerous mitochondrial proteins that were not detected or removed from the data due to stringent filtering parameters, may influence synaptic stability in vivo. Despite this, it is abundantly clear that proteomics and molecular genetics are powerful tools to identify candidates that may impact upon synaptic structure and function.

## Conclusions

The current study has demonstrated novel insights into how a ‘top-down’ approach may be utilised for identifying novel candidates that may modulate the stability of synaptic compartments in vivo. Our investigation has established that the morphological and biochemical properties of synaptic and non-synaptic mitochondria differ significantly across a multitude of mammalian species. Using molecular genetic tools at the *Drosophila* neuromuscular junction, manipulation in the expression several candidates that displayed increased abundance in synaptic mitochondria, resulted in selective alterations in synaptic, but not axonal morphology in vivo. Thus changes in mitochondrial protein expression may contribute to increased synaptic vulnerability in early molecular pathological processes during ageing and/or disease.

## Additional files

Additional file 1: Table S1. All proteins identified from synaptic and non-synaptic mitochondrial proteomic analyses. (XLSX 273 kb)

Additional file 2: Table S2. All proteins identified from synaptic and non-synaptic mitochondrial proteomic analyses with >2 fold-change, *p*≥0.05, ≥2 unique peptides. (XLSX 106 kb)

## Abbreviations

ATP: Adenosine triphosphate
BCA: Bicinchoninic acid assay
FASP: Filter aided sample preparation
HPLC: High performance liquid chromatography
HRP: Horseradish peroxidase
IPA: Ingenuity pathway analysis
M: Non-synaptic
NMJ: Neuromuscular junction
NS: Non-synaptic mitochondria
QFWB: Quantitative fluorescent western blotting
S: Synaptic mitochondria
SM: ‘light’ synaptic mitochondrial fraction
SM2: ‘heavy’ synaptic mitochondrial fraction
Syn: Synaptosomal
WT: Wild-type
